# Surface Engineering of NiTi via Ta_2_O_5_ ALD: Stability Assessment of Barrier Properties against Exposure to Simulated Physiological Solution

**DOI:** 10.1021/acsami.4c22646

**Published:** 2026-05-06

**Authors:** Julia Kolasa, Anna Taratuta, Barbara Rynkus, Julia Lisoń-Kubica, Karolina Wilk, Ada Orłowska, Maciej Krzywiecki, Jerzy Bodzenta, Karolina Szawiraacz, Karla Čech Barabaszová, Sylva Holešová, Anna Ziębowicz, Roman Major, Przemysław Kurtyka, Marcin Staniek, Marcin Basiaga

**Affiliations:** † Department of Biomaterials and Medical Device Engineering, Faculty of Biomedical Engineering, 49569 Silesian University of Technology, Roosevelta 40, 41-800 Zabrze, Poland; ‡ Institute of Physics - Centre for Science and Education, Silesian University of Technology, Konarskiego 22B, 44-100 Gliwice, Poland; § Institute of Metallurgy and Materials Science, 243566 Polish Academy of Sciences, Reymonta 25, 30-059 Kraków, Poland; ∥ Nanotechnology Centre, Centre for Energy and Environmental Technologies (CEET), VŠB - Technical University of Ostrava, 17. Listopadu 2172/15, 708 00 Ostrava, Czech Republic; ⊥ Department of Transport Systems, Traffic Engineering and Logistics, Faculty of Transport and Aviation Engineering, Silesian University of Technology, Krasińskiego 8, 40-019 Katowice, Poland

**Keywords:** NiTi shape memory alloy, atomic layer deposition (ALD), tantalum pentoxide, protective coatings, corrosion resistance, biocompatibility

## Abstract

Tantalum pentoxide (Ta_2_O_5_) coatings deposited via atomic layer deposition (ALD) have emerged as promising protective barriers for NiTi shape memory alloys in biomedical applications. This study investigates the influence of 800 ALD cycles on the structural, tribological, and electrochemical stability of NiTi substrates during long-term exposure to physiological environments. A conformal, amorphous Ta_2_O_5_ layer with a thickness of ∼65 nm was achieved, effectively sealing the substrate topography. Our findings reveal that while the coating initially maintains a hydrophilic character, prolonged incubation (60 days) in PBS solution triggers a transition to a hydrophobic state of 104 ± 3°, driven by the accumulation of Ca- and P-mineralized deposits. Tribological assessments demonstrate that the Ta_2_O_5_ overlayer enhances wear resistance and load-bearing capacity, despite localized microdelamination observed postexposure. Most notably, the ALD coating acts as a robust diffusion barrier, reducing the corrosion current density to 7.60 × 10^–8^ A/cm^2^ and maintaining a high polarization resistance even after 60 days of incubation. These results highlight the efficacy of Ta_2_O_5_ overlayers in providing long-term biostability and corrosion protection for NiTi-based implants in demanding physiological conditions.

## Graphical abstract

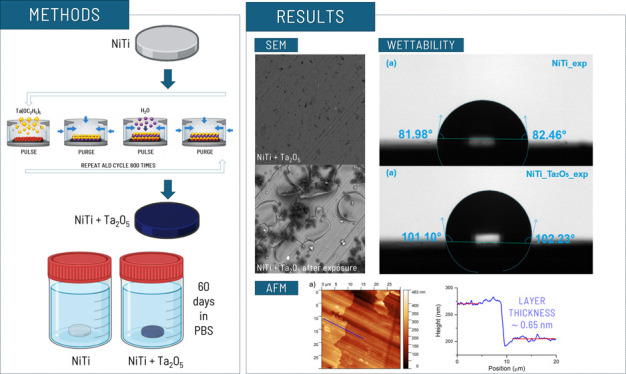

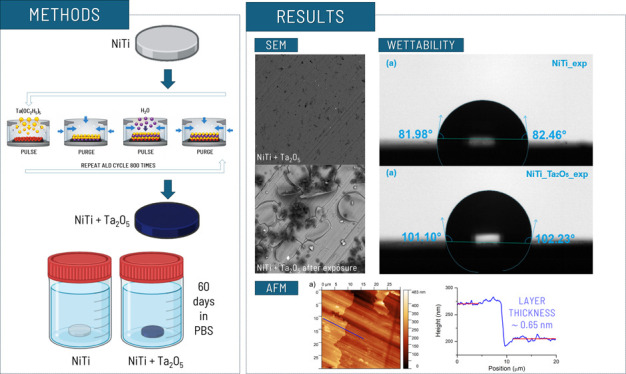

## 1 Introduction

Nickel–titanium (NiTi) alloys occupy an established position in contemporary biomedical engineering and are widely used in implants and medical devices such as vascular stents, guidewires, and orthodontic components.^1,2^ Their continued application is primarily driven by a combination of functional properties that are well aligned with the mechanical and physiological requirements of the human body. A defining characteristic of NiTi alloys is the shape memory effect, which allows a device to recover a predefined geometry in response to temperature changes.^3,4^ Complementing this behavior is superelasticity, enabling large reversible strains under mechanical loading without permanent deformation. Together, these properties support minimally invasive deployment and reliable in vivo performance. In addition, the relatively low elastic modulus of NiTi, compared with conventional metallic biomaterials, results in a more favorable mechanical compatibility with biological tissues, reducing stress shielding and improving functional integration.^5,6^ Despite its well-documented performance and established manufacturing and regulatory procedures, the long-term stability of NiTi in the human body still requires attention. The in vivo environment is electrochemically aggressive; it acts as a natural electrolyte, containing chloride ions and undergoing dynamic pH changes. Under such conditions, the surface of implants may degrade, which means that electrochemical processes play a key role in assessing the durability and safety of medical devices.^7,8^ Although NiTi has exceptional mechanical and functional properties, it faces specific challenges in the in vivo environment related to the surface of the material. A passive layer rich in titanium oxides (TiO_2_) naturally forms on it, protecting the alloy from direct contact with biological fluids. However, its stability under physiological conditions is limited, especially in the presence of chloride ions, variable pH values, and local degradation processes.^9,10^ As a result, local surface defects may appear, exposing nickel-containing phases. The release of Ni ions is a significant problem due to their potential to cause allergic reactions, cytotoxicity, and associated regulatory restrictions, making passivation control a key issue in the design and use of NiTi implants.^11–13^

To address these challenges, numerous surface modification strategies for NiTi have been investigated, including controlled chemical passivation,^14^ anodization to thicken oxide layers,^15,16^ and deposition of ceramic coatings such as metal oxides and nitrides.^17,18^ These approaches aim to create an effective diffusion barrier that limits direct interactions between the alloy substrate and the in vivo environment, thereby enhancing corrosion resistance and reducing nickel release. However, despite the reported improvements, these methods have not provided a definitive solution. In practice, the resulting layers often exhibit nonuniformity, susceptibility to microcracking, limited control over thickness, and insufficient long-term stability under in vivo conditions, indicating that reliable and durable surface stabilization of NiTi remains an unresolved materials engineering challenge. These limitations indicate that the key challenge is not only the choice of coating material but also the ability to create an ultrathin, uniform, and defect-free barrier layer with precisely controlled thickness on a complex metal substrate. Achieving such a layer requires a deposition method that enables the uniform growth and control of layer formation at the atomic scale. This need draws attention to techniques specifically designed for nanoscale barrier engineering such as atomic layer deposition (ALD).

ALD is an advanced technique characterized by its conformality, exceptional thickness control precision, and ability to produce ultrathin, defect-free coatings. Unlike conventional coating methods, where layer thickness and uniformity are difficult to control on irregular surfaces, ALD relies on self-limiting surface reactions, enabling repeatable and atomically precise film growth, even on complex topographies. These characteristics make ALD a particularly promising tool in biomaterial barrier engineering, where it is crucial to minimize direct interactions between the substrate and the aggressive in vivo environment, limit ion release, and improve corrosion resistance. In the context of implants and medical devices, ALD has found many applications, from oxide surface stabilization to nanoscale protective coatings and to the functionalization of materials that are difficult to coat with other methods. With its ability to deposit ultrathin, stable barriers, ALD offers a solution to problems such as coating heterogeneity, microcracks, and limited durability observed with traditional modification techniques.^19–22^

The choice of tantalum oxide (Ta_2_O_5_) as a barrier layer material is due to its favorable chemical and biological properties. Ta_2_O_5_ is characterized by high chemical stability and corrosion resistance in chloride-rich environments and over a wide pH range, which is particularly important in in vivo applications. In addition, tantalum oxide is biocompatible and is well tolerated by the body, which reduces the risk of local adverse reactions when in contact with tissues. Literature reports indicate the use of Ta_2_O_5_ in a biomedical context both as a surface-stabilizing layer for implants and as a component of functional coatings in diagnostic and therapeutic devices.^23–26^ In this work, Ta_2_O_5_ acts as a barrier layer to reduce ion transport and improve the durability of the NiTi surface, rather than as an active biological interaction, making it a reasonable choice for ultrathin ALD coatings.

Despite the demonstrated initial corrosion resistance of Ta_2_O_5_ coatings on NiTi, their long-term structural integrity and evolution in aggressive physiological environments remain largely unexplored. **The novelty of this work lies in the systematic investigation of the synergistic relationship between the ALD-derived Ta**_**2**_ **O**_**5**_ **nanostructure and its long-term interfacial behavior in simulated body fluids**. We hypothesize that the precise atomic-scale control of the Ta_2_O_5_ barrier not only provides superior electrochemical stability but also dictates a specific surface mineralization pathway that fundamentally alters the wettability and tribological response of the system. This study aims to bridge the gap between initial deposition characteristics and long-term in vitro durability, providing a mechanistic understanding of how Ta_2_O_5_ overlayers evolve under prolonged exposure to physiological stress.

## 2 Materials and Methods

NiTi alloy, also known as nitinol, was used as a substrate. 14 mm-diameter circular discs were laser-cut from a 0.8 mm-thick sheet and then polished to achieve Ra = 0.16 μm. Subsequently, the specimens were rinsed in pure 2-propanol and then in deionized water with an ultrasonic device for 15 min and dried in air. In the next step, a thin coating of Ta_2_O_5_ was applied using the ALD technique. Appropriately selected precursors were used for this: heated tantalum ethoxide Ta­(OC_2_H_5_)_5_ and water (H_2_O). The process was carried out at a temperature of 150 °C. The detailed values of times for both factors were 500 ms for pulses and 2 s for purging steps. The role of the transport and purification gas was played by nitrogen with a purity of 5.0 (N_2_) maintained at a constant flow of 200 cm^3^/min for both the precursor and the water. The samples were coated with a tantalum­(V) oxide coating obtained during 800 ALD cycles. The number of cycles and process parameters were determined on the basis of previous pilot studies.^27^ Each sample was immersed in 10 mL of phosphate buffer (PBS, pH 7.4) and incubated at 37 °C for 60 days under static conditions. This static immersion protocol was employed to simulate the chemical environment within vascular recirculation zones and to facilitate a cumulative assessment of the coating’s barrier properties. By avoiding solution renewal, a “worst-case scenario” of local ionic accumulation was established, which is critical for evaluating the long-term stability of the Ta_2_O_5_ layer in low-flow hemodynamic regions.

In order to maintain a uniform form of presentation of the results of testing samples prepared in accordance with the procedure described above, the following nomenclature has been considered:

- *NiTi*: initial-state sample made of NiTi alloy
- *NiTi_exp*: initial-state sample made of NiTi alloy after exposure to the PBS solution
- *NiTi_Ta*_2_ *O*_5_: NiTi alloy coated with tantalum­(V) oxide obtained using the ALD technology during 800 cycles
- *NiTi_Ta*_2_ *O*_5_ *_exp*: NiTi alloy coated with tantalum­(V) oxide obtained using the ALD technology during 800 cycles after exposure to the PBS solution

### 2.1 Surface Morphology (SEM)

The surface assessment was carried out using a scanning electron microscope (SEM) (TESCAN VEGA, Brno, Czech Republic) with BSE and SE detectors at an energy of 10 keV. As part of the surface evaluation, the chemical composition of the surface layer was analyzed by energy dispersive spectroscopy (EDS) using an Xplore EDS detector (Oxford instruments, Oxford, UK) coupled to a SEM. Measurements were performed at 20 keV and 3 nA.

### 2.2 X-ray Diffraction Analysis

X-ray diffraction (XRD) analysis was conducted to determine the phase compositions of the base material (NiTi alloy) and the surface-modified material (NiTi coated with Ta_2_O_5_). The aim of the analysis was to identify the crystalline phases present on the sample surface before and after the surface modification process. Measurements were performed using a D8 Discover diffractometer (Bruker) configured for localized point analysis. The system allows automated acquisition of diffraction patterns from user-defined measurement points on the sample surface using a focused and filtered X-ray beam. The incident beam was conditioned using a polycapillary optic acting as a collimation and focusing system, enabling microarea analysis with enhanced beam intensity and spatial resolution. The diffractometer was equipped with a cobalt anode X-ray source (Co Kα_1_ radiation, λ = 1.78897 Å), and diffraction data were collected using a LynxEye stripe detector. The measurements were carried out in the 2θ range of 20°–80°. Phase identification was performed by using the DIFRAC.EVA software package (Bruker) in combination with the Powder Diffraction File database (PDF-4+, ICDD).

### 2.3 X-ray Photoelectron Spectroscopy

The surface chemical analysis was done utilizing the X-ray photoelectron spectroscopy (XPS) method. The setup was installed in an ultrahigh-vacuum multichamber experimental system (base pressure: 8.8·10^–9^ Pa) and fitted with a PREVAC EA15 hemispherical electron energy analyzer (using a 2D-MCP detector). For sample excitation, a monochromated X-ray beam (PREVAC dual-anode XR-40B source, RMC50 monochromator; Al–Kα excitation line with energy: 1486.60 eV) was used at 120 W operating power. For the full survey scan (energy step: 0.9 eV), the pass energy was set to 200 eV, while for the detailed high-resolution scan of particular energy regions of interest, a pass energy of 100 and 0.07 eV step were applied. Measurements were made with a normal takeoff angle and the curved (0.8 × 25 mm^2^) analyzer exit slit. The binding energy (BE) scale was calibrated with respect to the Au 4f7/2 (84.0 eV) region of the gold-covered sample placed at the same sample stage.^28^ Greczynski and Hultman^29^ procedures were applied to avoid possible charging-related distortions. Recorded spectra were postprocessed using CASA XPS software (version 2.3.25) with the application of built-in algorithms and relative sensitivity factors. Shirley’s function was applied for the background subtraction.

### 2.4 Fourier Transform Infrared Spectroscopy

The chemical structure samples were evaluated by infrared (IR) spectroscopy. The IR spectra were collected using the Nicolet iS50 FT-IR spectrometer (ThermoScientific) with a DTGS detector on a Smart Orbit ATR accessory. The IR spectra were measured by the ATR (attenuated total reflectance) technique on a single-reflection diamond ATR crystal. The measurement parameters were as follows: spectral region: 4000–400 cm^–1^, spectral resolution: 4 cm^–1^; 64 scans; Happ-Genzel apodization.

### 2.5 Atomic Force Microscopy with Scratch

An XE-70 atomic force microscope (Park Systems) was used for the determination of Ta_2_O_5_ film thickness, analysis of local stiffness, and analysis of the friction force between the layers and AFM probe tips.

Ta_2_O_5_ film thickness was determined from topographical profiles measured across the film edge. These measurements were carried out in noncontact mode using Tap300DLC probes (Budget Sensors).

Local stiffness was analyzed based on load–displacement curves measured in nanoindentation mode.^30^ The method is based on the measurement of load–displacement curves for one cycle of loading and unloading. It works for a variety of axisymmetric indenters (AFM probe tips). Detailed description of force–distance measurements utilizing AFM can be found in the literature.^31^ The initial local stiffness can be determined from the maximum slope of the unloading curve. In these measurements, a Tap300DLC probe was used.

The influence of Ta_2_O_5_ films on probe tip–sample friction was based on the analysis of images obtained by lateral force microscopy. This method utilizes contact AFM imaging. Lateral forces cause the beam to twist, and this “twisting” signal conveys information about local friction forces. In these measurements, a ContAl-G probe (Budget Sensors) was used.

### 2.6 Topography and Microroughness

Surface topography measurements were carried out using a Leica DCM 8 optical profilometer operating in confocal mode with green light and a 20× objective. The examined surface area was 880 × 660 μm^2^. Data processing and surface evaluation were performed with Leica Map Premium 9 software. For each experimental variant, three surface regions were selected at random for analysis. From each region, three surface profiles were extracted. To provide a comprehensive morphological characterization, four key roughness parameters were determined: the arithmetical mean height of the surface (Sa), maximum height of the surface (Sz), arithmetical mean deviation of the profile (Ra), and maximum height of the profile (Rz).

### 2.7 Wettability and Surface Free Energy

Surface wettability studies were carried out by using the sessile drop method. Contact angle (Θ) measurements were performed with an Attension Theta Flex optical tensiometer (Biolin Scientific) and analyzed by using OneAttension software. Distilled water and diiodomethane were used as test liquids, with a droplet volume of 1.5 mm^3^. The measurement started 15 s after droplet deposition and lasted 60 s. Based on the average contact angles measured for water and diiodomethane, the surface free energy (SFE) was determined using the Owens–Wendt–Rabel–Kaelble (OWRK) method. The studies were conducted for samples in the initial state as well as after exposure to the PBS solution.

### 2.8 Tribology

The abrasive wear resistance of the coating was tested using the ball-on-disc method with an Anton Paar TRB tribometer, applying a load of 0.5 N. A ball made of steel AISI 440-C (Ra ≤ 0.05 μm) with a diameter of 6 mm was used as a counter sample in the friction pair with the tested material, and the grip with the tested material rotated at a speed of 2 cm/s, with 1 Hz frequency and 1000 cycles. The environmental parameters were 26% humidity and 23 ± 1 °C. The measurements were carried out by setting the distance traveled by the ball to 21 m. The resistance to movement was determined during technically dry friction, TDF (test of dry friction). In the first step, measurements of the weight samples were conducted before tribological testing. Next, after conducting tribological tests to assess surface morphology and measurement of weight and analyzing the geometric structure of the surface of the friction mark, a Leica DCM8 optical profilometer with interferometric mode (with LeicaMap software at 20× magnification) was used. A single measurement covered an area of 840 × 630 μm^2^. At the same time, the wear area and wear depth were evaluated to quantitatively determine the material’s resistance to abrasive wear.

### 2.9 Corrosion Studies

Cyclic potentiodynamic polarization studies were performed using an Autolab PGSTAT302N potentiostat with Nova 2.1 software. A standard three-electrode system was used for the tests, consisting of a reference electrode, which was a silver chloride electrode (Ag|AgCl in 3 M KCl), a counter electrode in the form of a platinum wire, and a working electrode, which was the test sample.

The tests of each variant began with the determination of the open-circuit potential E_ocp_ over a period of 3600 s. The polarization curves were recorded from the initial potential value E = E_ocp_ – 200 mV at a scanning rate of 0.3 mV/s. Polarization curves were then recorded until a potential value of 2 V or a current density of 1 mA/cm^2^ was reached, followed by a change in the direction of polarization. The tests were carried out in a PBS (phosphate-buffered saline) solution at a temperature of 37 ± 1 °C.

## 3 Results and Discussion

### 3.1 Surface Morphology

The NiTi substrate was characterized by a surface morphology typical of mechanically processed materials; numerous parallel grooves and isolated burrs formed during grinding and mechanical polishing were observed on the surface (Figure 1S). The coating deposited by the ALD method did not affect the surface morphology, preserving its original character (Figure 1) The presence of the Ta coating was confirmed by EDS analysis (Figure 2). The produced coating was homogeneous and continuous throughout the entire sample volume.

**Figure 1:**
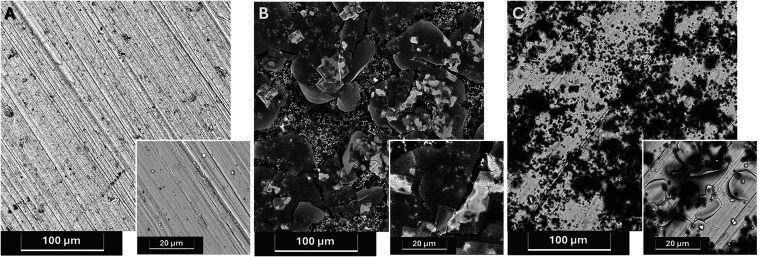
Surface morphology of modified samples in SEM: (A) −NiTi_Ta_2_O_5_. (B) −NiTi_Ta_2_O_5__exp with visible Ca and P deposits on the surface. (C) −NiTi_Ta_2_O_5__exp cleaned with demineralized water.

**Figure 2:**
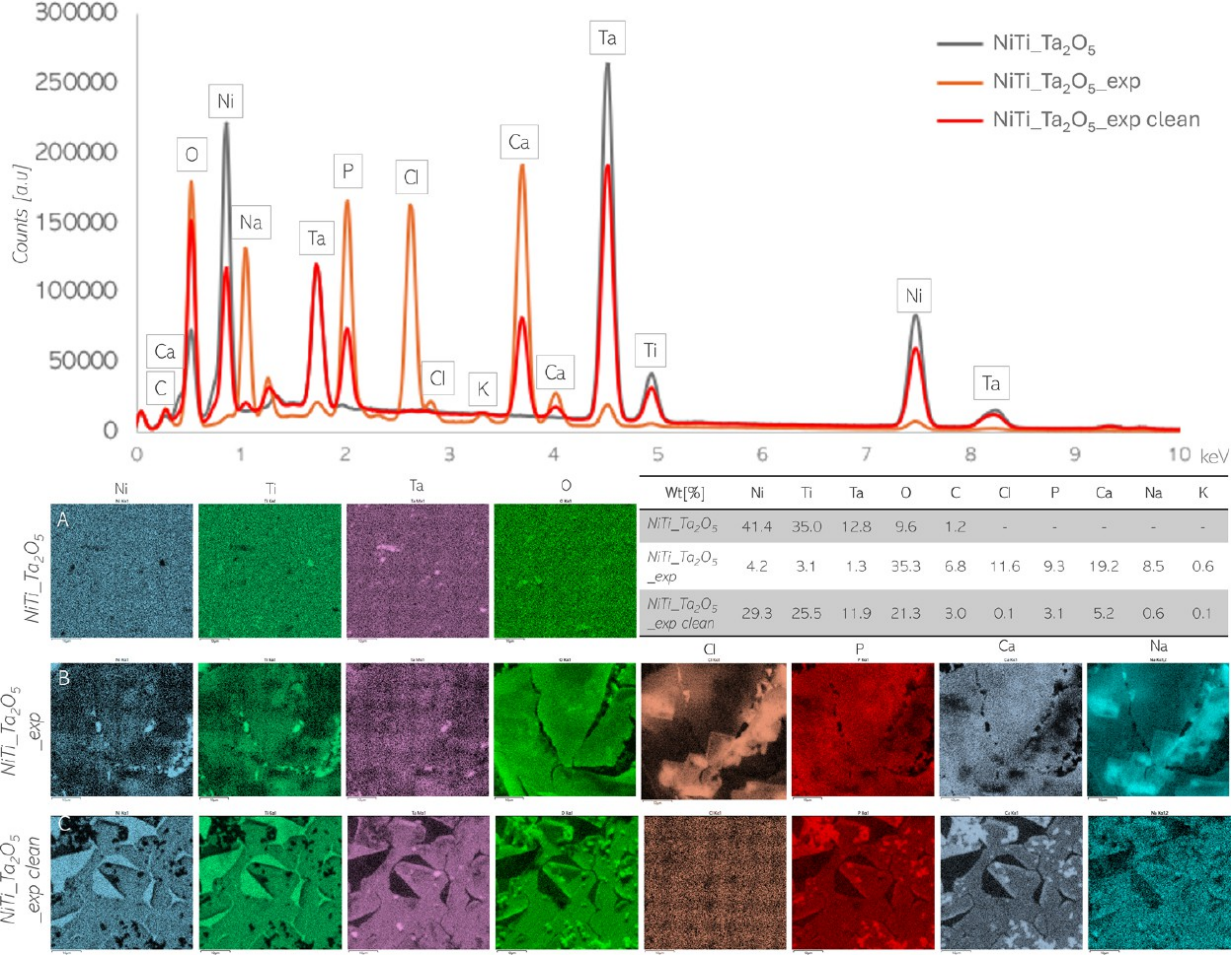
Example EDS spectra for the material coated with Ta_2_O_5_ nanocoating for samples: (A) −NiTi_Ta_2_O_5_, (B) −NiTi_Ta_2_O_5__exp, and (C) −NiTi_Ta_2_O_5__exp after cleaning, with the determined % share of individual elements and the element distribution map.

Observations carried out for samples after exposure to an environment simulating tissue conditions (PBS solution) revealed significant aggregation of Ca- and P-containing compounds on the sample surface (Figure 2). A deposit with a crystalline cracked structure covered the entire surface, forming a barrier layer. This phenomenon was not observed for the unmodified surface, where minor surface contamination with a spherical structure was observed. Cleaning of the surface with demineralized water resulted in substantial removal of the deposits. However, the presence of a residual layer composed of elements originating from the PBS solution was still detected. In addition, numerous cracks within the formed Ta coating as well as local delamination of the coating were observed on the surface.

### 3.2 X-ray Diffraction Analysis

The XRD patterns recorded for both the uncoated NiTi alloy and the Ta_2_O_5_-coated samples (Figure S2) are dominated by diffraction peaks corresponding to the B2 austenitic phase of NiTi, which is characteristic for the NiTi substrate and remains clearly detectable after the deposition of the coating layer. Minor contributions from the B19′ martensitic phase were observed. This confirms that the crystalline structure of the substrate is preserved following the surface modification process.

A reduction in the intensity of the NiTi diffraction peaks is observed after the deposition of the Ta_2_O_5_ layer. This effect can be attributed to the attenuation of both the incident and diffracted X-ray beams by the thin coating, which partially limits X-ray penetration and consequently decreases the diffracted signal originating from the underlying substrate. Such behavior is commonly observed for thin surface coatings analyzed using conventional XRD geometry. No additional diffraction peaks associated with crystalline Ta_2_O_5_ phases were detected in the diffraction patterns. This indicates that the deposited Ta_2_O_5_ layer is amorphous, which is consistent with the structural characteristics typically reported for atomic layer deposition (ALD) films synthesized at relatively low deposition temperatures.

Furthermore, no measurable shifts in the NiTi peak positions or the formation of additional phases was observed after coating deposition. This suggests that the ALD process does not induce phase transformations, interdiffusion, or crystallographic changes within the NiTi substrate. The results therefore confirm that the applied coating process is nondestructive and preserves the crystallographic integrity of the NiTi alloy, while introducing a uniform surface modification layer.

### 3.3 X-ray Photoelectron Spectroscopy

The surface chemical composition was investigated with the surface-sensitive XPS method. The results are shown in Figure 3. For the following discussion, in the case of panels (a, e), the top row (green points) corresponds to the as-received sample, while bottom spectra (red points) represent spectra from the processed sample. Survey scans of the as-received sample (Figure 3a top spectrum) revealed the expected tantalum oxide components, i.e., Ta and O, as represented by the main core-level lines Ta 4f and O 1s, respectively. The minor C 1s region is visible as a result of the adventitious carbon presence. After sample processing, Ta and O signals are significantly reduced, while the Ti signal emerges. As a result of processing, Ca and P signals appear. The analysis of high-resolution spectra revealed further consequences of sample processing.

**Figure 3:**
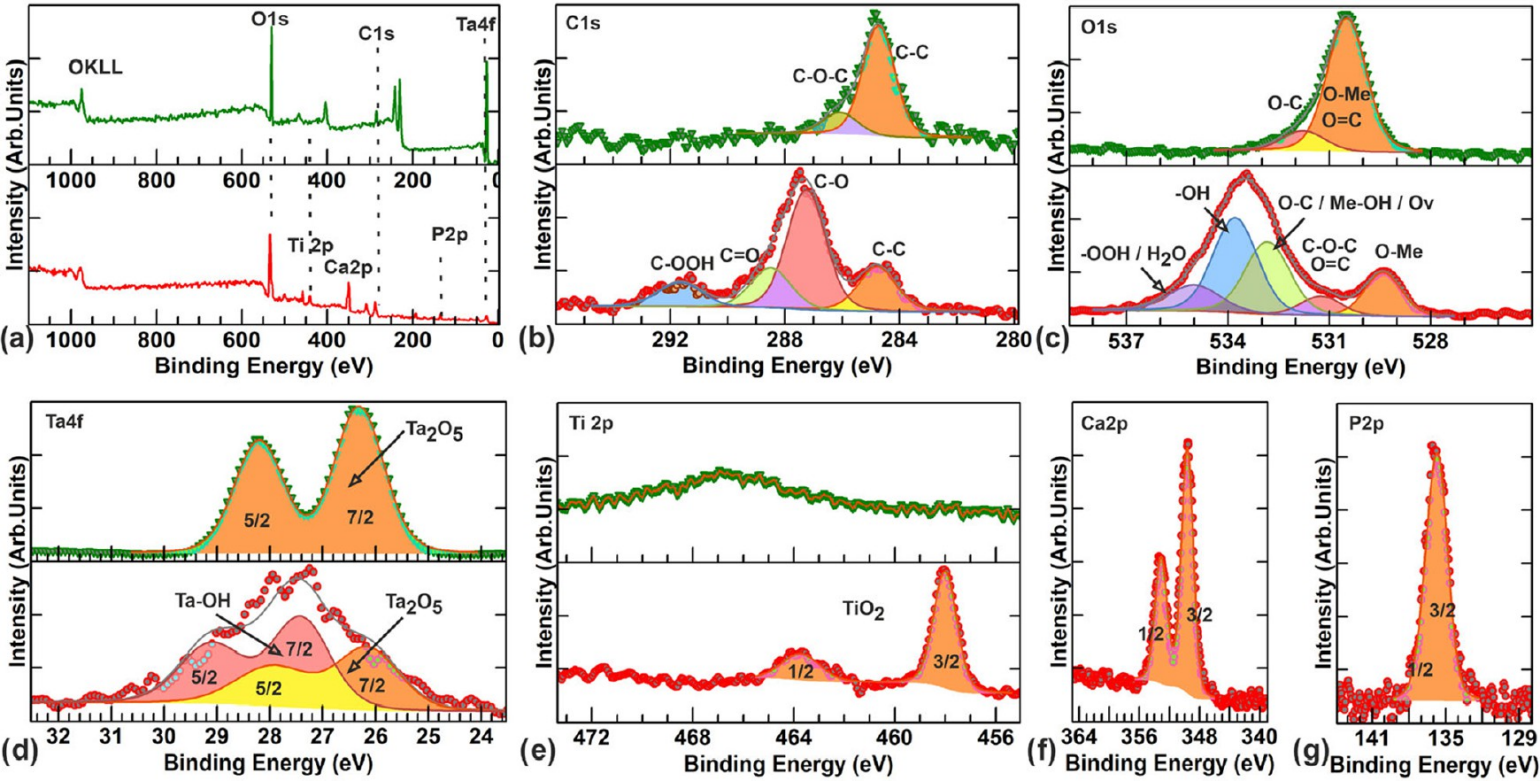
(a) Survey spectra of the examined samples and high-resolution energy regions: (b) C 1s; (c) O 1s; (d) Ta 4f, (e) Ti 2p; (f) Ca 2p, and (g) P 2p. For panels (a–e), the top row corresponds to the as-received sample, while the bottom row represents the spectra from the processed sample. Ca 2p and P 2p regions are shown only for the processed sample.

In the case of the C 1s region (Figure 3 b), the minor carbon contribution to the surface is mainly represented by the C–C and C–O–C components at 284.8 and 286.0 eV, respectively. For the processed sample, the C–C signal is still visible; however, it is being dominated by the C–O component at ∼287.0 eV and accompanied by C=O and COOH components at 288.5 and 291.0 eV, respectively.^32^ The analysis of the O 1s region confirms the carbonaceous contribution composition, as represented by the O–C and C–O–C components. As expected, in the case of the as-received sample, the dominant component is, however, the O-Me one, which represents oxygen bonded to the tantalum counterpart in the oxide lattice.^33^ This component also possesses possible small contributions originating from the O=C configuration. For the processed sample, the O–C component is being slightly shifted toward high binding energies as a result of additional contributions from Me–OH (most likely Ta–OH^32,34^ ) configurations and the oxygen vacancy (Ov)-rich disturbed oxide lattice.^35^ Next, −OH and H_2_O components can be detected as a clear result of sample processing residuals.

The Ta 4f region recorded for the as-received sample exhibits a classical nearly stoichiometric Ta_2_O_5_ configuration^36^ with classical spin–orbit splitting of 7/2 and 5/2 components (Figure 3d top). No suboxides are detectable, which points to successful ALD layer deposition. After processing, the spectrum is much more complicated. The Ta_2_O_5_ signal is still recognizable; however, a new doublet at ∼27.2 eV (7/2 component position) appears and can be assigned to the degraded oxide mixed with Ta–OH compounds.^34^ The high impact of processing on the surface composition of the examined samples is further confirmed by the Ti 2p energy region (Figure 3e). As one can see in Figure 3, for the as-received sample, the titanium signal is not visible. The situation changes drastically after the processing of the sample surface upon which a strong Ti signal appears. The structure of the signal, i.e., energy position at ∼457 eV (for the 2p 3/2 component) and strong symmetry of the 1/2 component, points to the high level of stoichiometry in the TiO_2_ configuration. Ca 2p and P 2p regions are shown only for the processed sample since no signal can be detected for the as-received sample. Ca 2p (Figure 3f) presents fine resolved spin–orbit splitting and an energy position close to values obtained for CaCO_3_ science.^37^ For the P 2p region, spin–orbit splitting is barely noticeable (components are very close to each other) and the energy position can indicate a metal-phosphate or P_2_O_5_ compound.^36,38^ Since the phosphate compound shall be significantly visible also in metal-originating region, i.e., Ta 4p or Ti 2p (and it is not), one can conclude that the P_2_O_5_ is more probable. However, the appropriate component in O 1s can be hard to detect due to carbon and phosphorus electronegativity values: with this respect, the O–P components will most likely overlap with O=C ones. Nevertheless, in both cases (Ca and P), the signal can be assigned to the postprocessing residuals.

### 3.4 Fourier Transform Infrared Spectroscopy

FTIR spectra of Ta_2_O_5_ coatings deposited by atomic layer deposition on a NiTi alloy substrate using the precursor shown in Figure 4, tantalum­(V) ethoxide, allow for unambiguous determination of the chemical composition and the degree of ordering of the oxide structure of the obtained layers. In all analyzed samples, very weak and wide bands in the range of 4000–3000 cm^–1^ were recorded, which can be attributed to the tensile vibrations of the hydroxyl–OH groups. Their presence is a consequence of the nature of the ALD process carried out at a relatively low temperature, which favors the retention of surface Ta–OH groups and the adsorption of moisture from the atmosphere after the deposition process is completed. The bands around 2920 and 2850 cm^–1^ are attributed to asymmetric and symmetric stretching vibrations of C–H (CH_3_ and CH_2_ groups) followed by the deformation band of C–H at 1430 cm^–1^, which indicates the presence of an unreacted tantalum precursor caused by the uneven effect of ALD deposition. Based on the band intensities, the biggest amount of precursor residue is in the sample 6_NiTi_Ta_2_O_5_. The most characteristic features of FTIR spectra are observed in the range of low wave numbers, typical of the lattice vibrations of metal oxides. In all samples, there is a distinct absorption band around 1110 cm^–1^, which is attributed to the vibrations of the Ta–O terminal groups.^39^ This band can be overlapped by the C–O stretching band of the ethoxy group. The intensity of this band increases with the increasing number of ALD cycles, which is a direct result of the increase in coating thickness and the progressive condensation of the oxide structure. At the same time, the observed band around 750 cm^–1^ corresponds to the tensile vibrations and deformation of Ta–O bonds in TaO_6_ polyhedra, confirming the formation of a typical Ta_2_O_5_ coordination network. The increase in the intensity of this band in samples with a higher number of cycles indicates an increasingly homogeneous and continuous layer, in which the signal input from the NiTi substrate is effectively attenuated.

**Figure 4:**
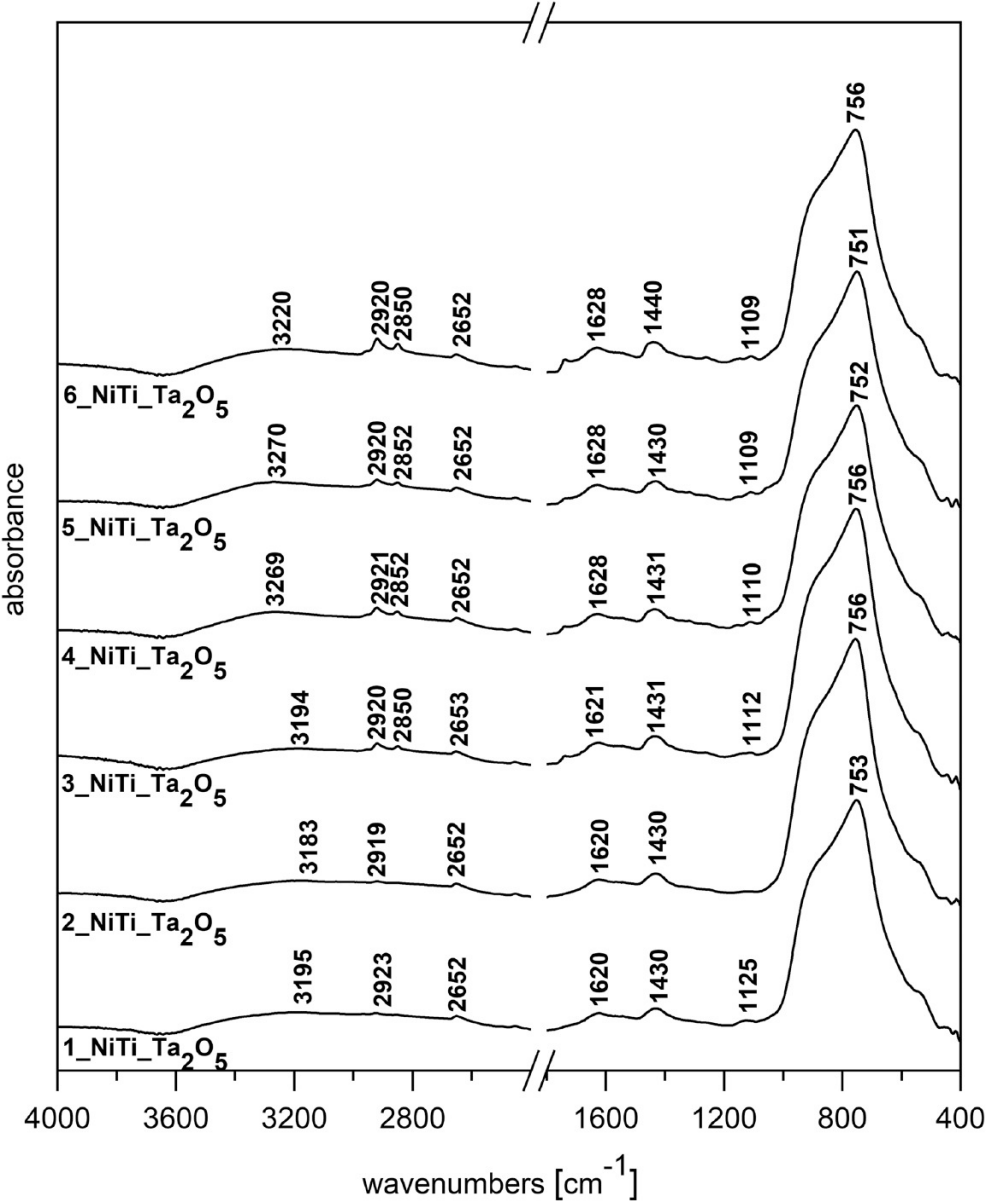
FTIR spectra of six Ta_2_O_5_ coatings deposited by ALD on a NiTi substrate using a tantalum­(V) ethoxide precursor after 800 deposition cycles.

The absence of distinct bands characteristic of nickel and titanium oxides suggests that the Ta_2_O_5_ coating effectively covers the NiTi substrate, creating a barrier that separates the substrate from the measurement environment. This is a direct proof of the high continuity and tightness of the ALD layers. The invariability of the positions of the bands with a simultaneous increase in their intensity with the number of ALD cycles indicates that the process leads to a repetitive growth of the chemically homogeneous, amorphous Ta_2_O_5_ layer, without significant changes in the chemical composition and local environment of the tantalum atoms.

### 3.5 Atomic Force Microscopy

Topographical images of the Ta_2_O_5_ layer edge with a high profile are shown in Figure 5. The edge was created by scratching off a portion of the layer. Based on the height profile shown in Figure 5b, the layer thickness was estimated to be 65.31 ± 0.21 nm. This value was obtained as the difference between the two levels, marked with horizontal red lines in the figure.

**Figure 5:**
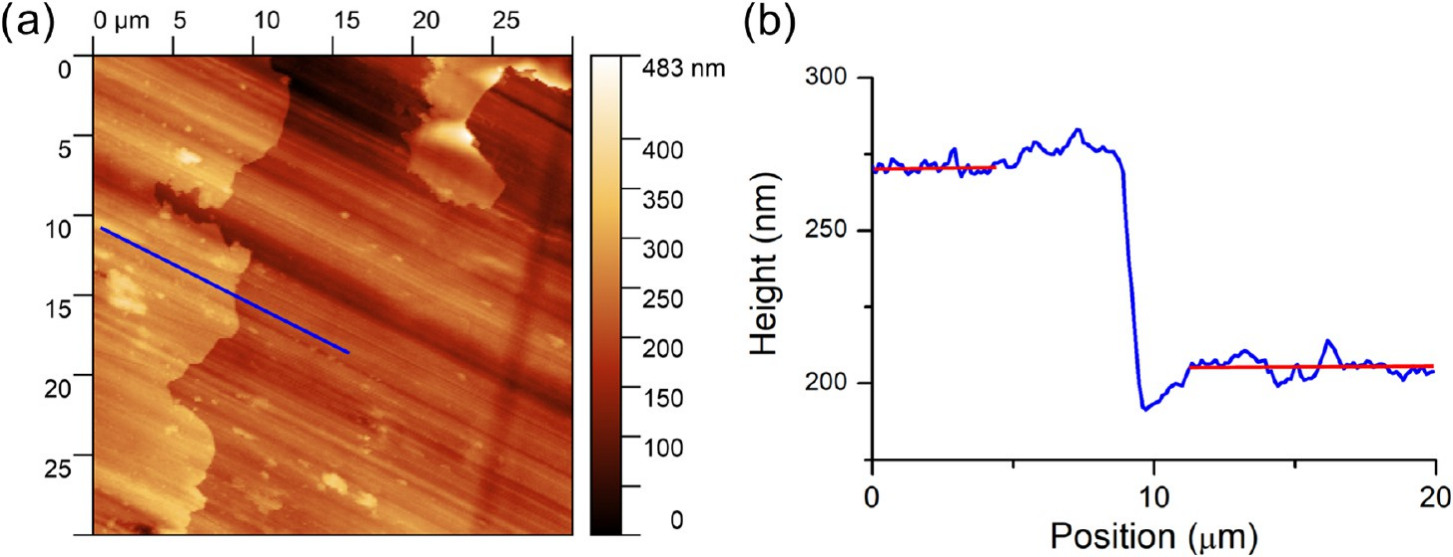
Topographic image of the layer edge (a) with the extracted height profile along the blue line (b). Horizontal red lines were fitted to the flat parts of the profile to determine the layer thickness.

As was stated in the section Materials and Methods, subsection AFM, local stiffness can be determined from the unloading (retract) part of the F–D curve. Such curves for the NiTi_Ta_2_O_5_ (curves 1–4) and NiTi_Ta_2_O_5__exp (curves 5–8) samples are shown in Figure 6. For the NiTi_Ta_2_O_5_ layers, the calculated stiffnesses range from 0.195 to 0.201 N/m, with three of the four values being close to the upper limit. The results for NiTi_Ta_2_O_5__exp range from 0.153 to 0.181 N/m, and the results are evenly distributed throughout the range. Therefore, the layers are softer after exposure, and their parameters differ at different positions.

**Figure 6:**
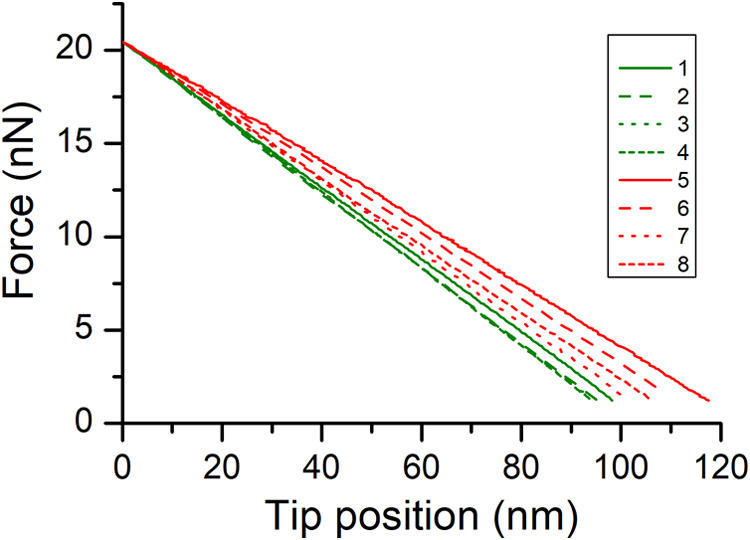
Unloading (retract) parts of force–distance curves measured at various positions on NiTi_Ta_2_O_5_ (curves 1–4) and NiTi_Ta_2_O_5__exp (curves 5–8) samples’ surfaces.

The last AFM measurements concerned the friction forces between the probe and the sample surface during scanning. Images in Figure 7 show calculated differences in lateral force signals measured for two scanning directions. Images correspond to the NiTi_Ta_2_O_5_ (a) and NiTi_Ta_2_O_5__exp (b) layers. Except for regions with large geometric structures (visible in topographic images), the calculated signal difference varies within small ranges. Frequency distributions of signals calculated for the green- and red-marked regions in Figure 7a,7b are shown in Figure 7c. It can be seen that lateral (friction) forces for the NiTi_Ta_2_O_5_ sample are smaller and around one value. These forces for NiTi_Ta_2_O_5__exp are about three times larger and vary over a relatively wide range. The mean values of the signals corresponding to the lateral forces were 0.125 ± 0.046 and 0.352 ± 0.071. However, it should be clearly stated that these values do not indicate the actual friction coefficients.

**Figure 7:**
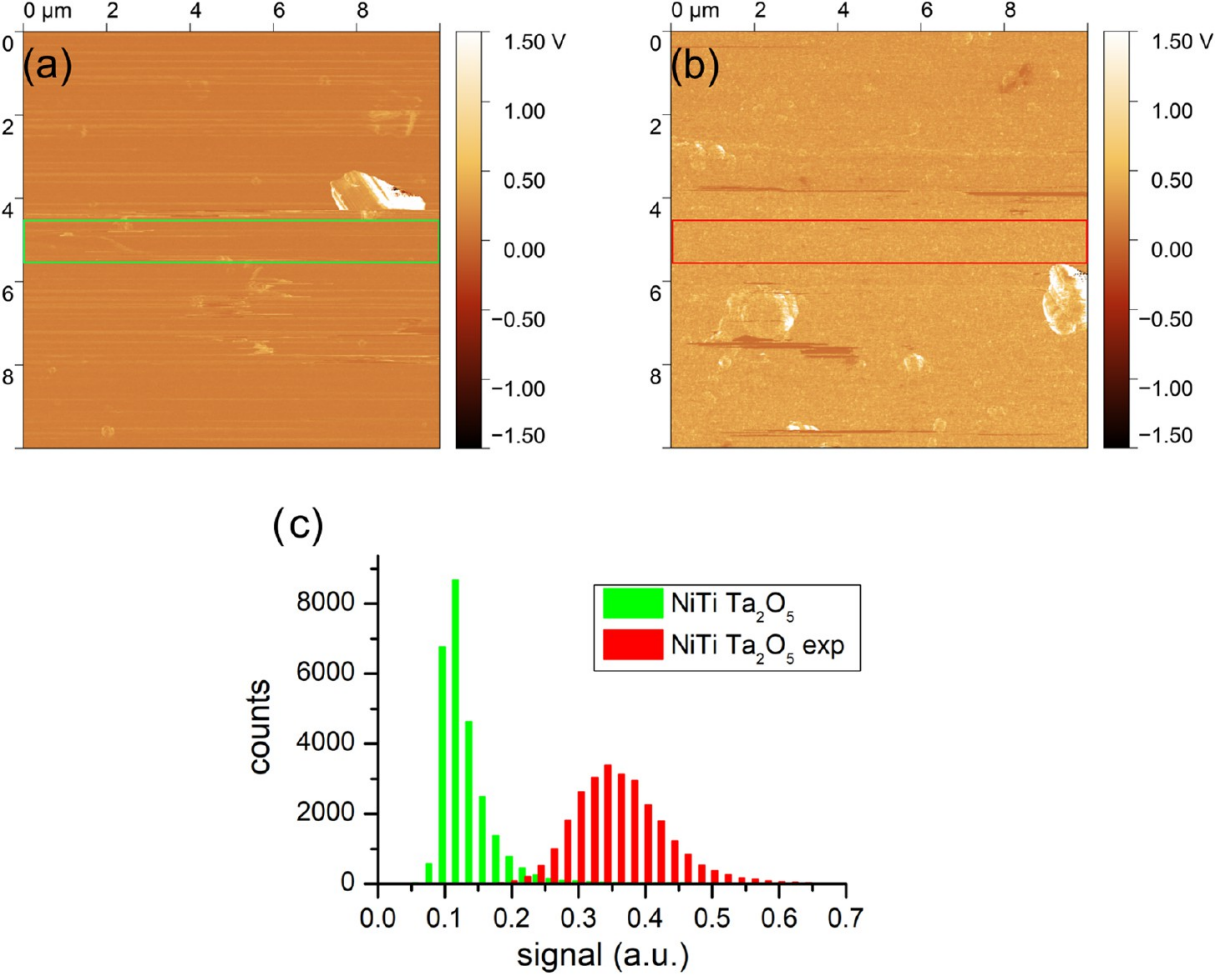
Difference of lateral force signals measured at two scanning directions for NiTi_Ta_2_O_5_ (a) and NiTi_Ta_2_O_5__exp (b) and frequency distributions of signals calculated for marked areas (c).

### 3.6 Topography and Microroughness

Topographical analysis revealed that the deposition of NiTi_Ta_2_O_5_ (Figure 9a) results in a subtle reduction of both average (Sa)- and height-based (Sz) roughness parameters compared to the baseline NiTi alloy (Table 1, Figure 8a). This phenomenon stems from the high fidelity of the ALD process and the excellent surface mobility of the utilized precursors.^40^ The atomic layer growth mechanism facilitates the preferential filling of nanometric defects and mechanical polishing marks, leading to the so-called “nanoscale smoothing effect.” Such homogenization of the surface profile is critical for ensuring the continuity of the dielectric barrier across the entire complex geometry of the implant.

**Figure 8:**
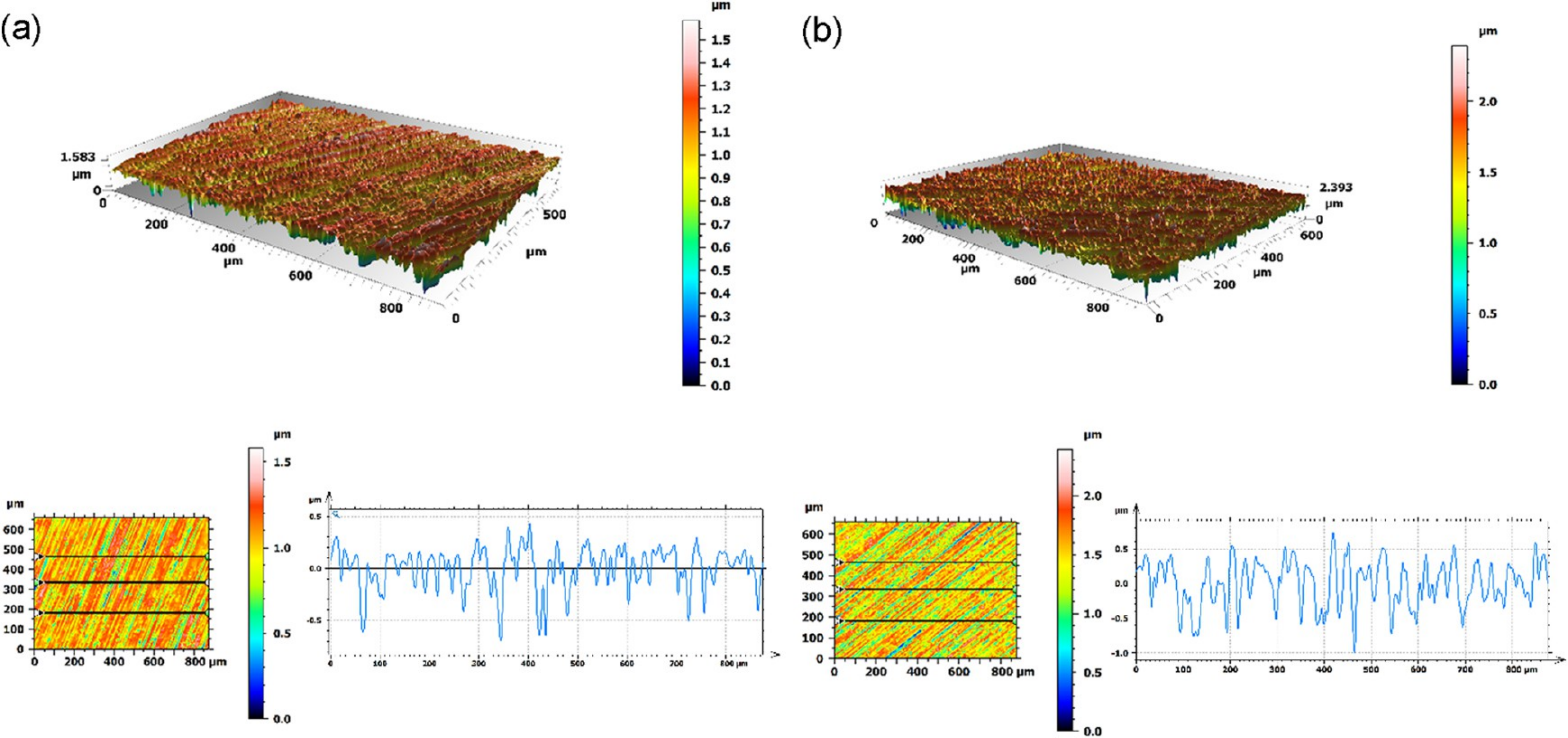
Surface topography and cross-sectional profiles of the uncoated material (a) NiTi and (b) NiTi_exp.

**Table 1:** Surface Roughness Parameters (Sa, Sz, Ra, Rz) of Uncoated NiTi and Ta_2_O_5_-Coated Samples before and after 60-Day Incubation in the PBS Solution

	Sa [μm]	Sz [μm]	Ra [μm]	Rz [μm]
NiTi	0.193 ± 0.008	1.561 ± 0.174	0.160 ± 0.020	1.185 ± 0.162
NiTi_exp	0.323 ± 0.064	2.489 ± 0.624	0.266 ± 0.037	1.866 ± 0.225
NiTi_Ta_2_O_5_	0.178 ± 0.013	1.371 ± 0.113	0.140 ± 0.016	1.024 ± 0.149
NiTi_Ta_2_O_5__exp	0.237 ± 0.047	1.604 ± 0.131	0.163 ± 0.029	1.159 ± 0.200

The implementation of a 60-day static incubation test enabled the simulation of a “worst-case scenario”, corresponding to vascular zones of restricted blood flow and stagnation. Under these conditions, the lack of solution exchange promotes localized ion accumulation and subsequent acidification of the microenvironment at the solid–liquid interface. For unprotected NiTi specimens, this prolonged exposure resulted in a drastic destabilization of the surface morphology (Figure 8b). The significant surge in the Sz and Sa parameters serves as clear evidence of progressive chemical degradation and the initiation of localized corrosive processes. These findings suggest that the native oxide layer on nitinol does not provide an adequate barrier under long-term physiological stagnation, which may lead to the exposure of the metallic matrix and the subsequent deleterious release of nickel ions.^41^

In stark contrast to the uncoated substrate, the Ta_2_O_5‑_coated system exhibited exceptional morphological stability (Figure 9b). Following the two-month incubation period, the roughness parameters of the coated samples remained at levels comparable to the initial baseline of the smart alloy. The minor alterations in topographical parameters observed after exposure should be interpreted not as coating degradation but rather as the result of phosphate buffer species adsorption onto the chemically inert and stable oxide surface. This confirms that the Ta_2_O_5_ layer acts as a robust diffusion barrier, preserving the structural and chemical integrity of the underlying NiTi substrate even under extreme physiological stress.

**Figure 9:**
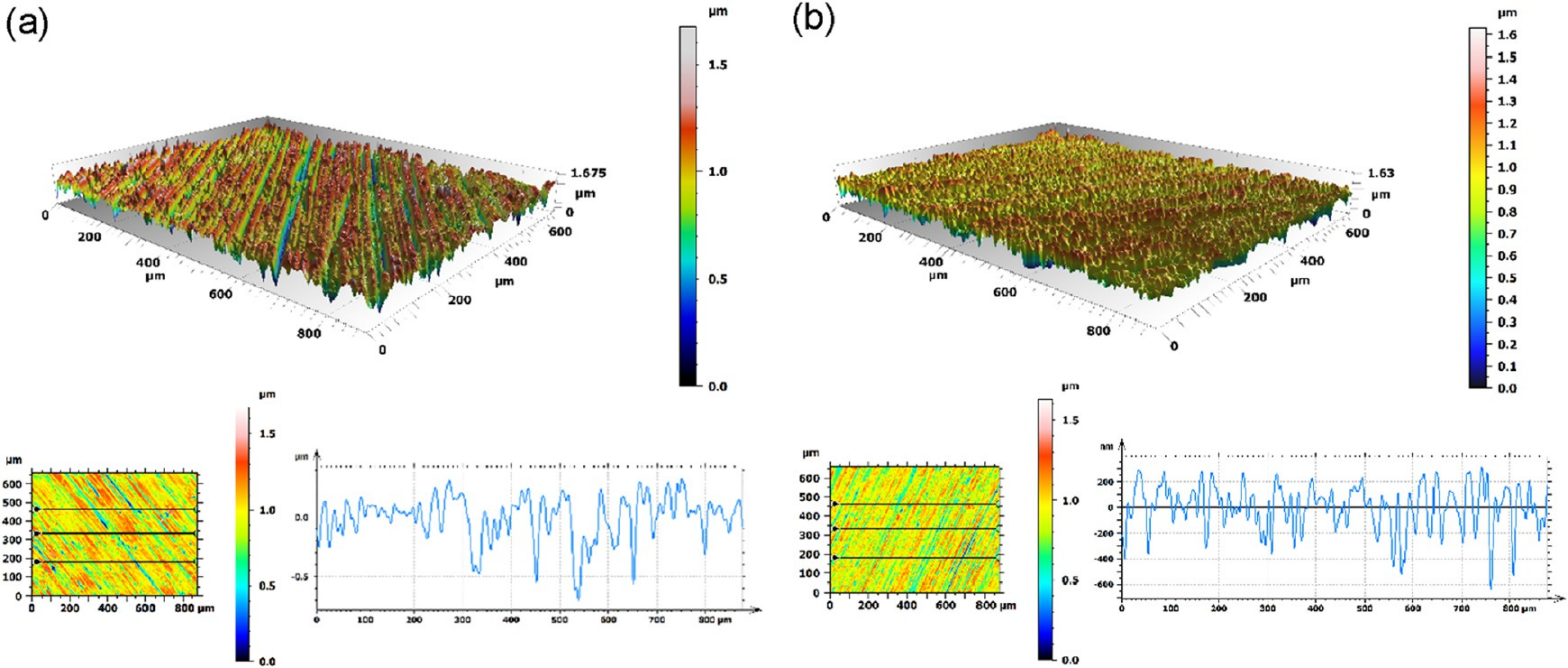
Surface topography and cross-sectional profiles of the ALD Ta_2_O_5_-coated samples (a) NiTi_Ta_2_O_5_ and (b) NiTi_Ta_2_O_5__exp.

### 3.7 Wettability and Surface Free Energy

The initial NiTi surface exhibited contact angles of 54 ± 4° for water and 54 ± 1° for diiodomethane, indicating a hydrophilic character with a relatively high total SFE of 51.45 mN/m and a significant polar component. After PBS exposure, the NiTi_exp samples showed a notable increase in the water contact angle to 83 ± 1° (Figure 10a) and diiodomethane (61 ± 3°, Figure 10b), while the surface remained hydrophilic; however, the total SFE decreased markedly to 32.69 mN/m, mainly due to a reduction of the polar component. The NiTi_Ta_2_O_5_ sample in the initial state also showed hydrophilic behavior, with water and diiodomethane contact angles of 69 ± 5° and 54 ± 2°, respectively, and a total SFE of 42.17 mN/m. In contrast, the NiTi_Ta_2_O_5__exp surface exhibited a pronounced increase in the contact angles for both water (104 ± 3°, Figure 11a) and diiodomethane (68 ± 3°, Figure 11b), indicating a definitive transition to hydrophobic behavior. This was accompanied by a substantial decrease in the total SFE to 24.16 mN/m and an almost complete loss of the polar component. The observed reduction in SFE reflects a spontaneous thermodynamic stabilization of the interface through the adsorption of low-energy species, effectively lowering the surface’s chemical potential. The marked reduction in SFE and the almost complete loss of the polar component after incubation in PBS are consistent with the spectroscopic results. Kilpadi’s studies established a direct correlation between the degradation of surface energy and the presence of adsorbed phosphorus- and carbon-containing species.^42^ This process involves chemical shielding of surface hydroxyl groups by phosphate ions and adventitious hydrocarbons, which fundamentally alters the interface’s ability to engage in polar interactions, thereby increasing the water contact angle. The detailed results for the contact angles and the calculated surface free energy components are summarized in Table 2. Overall, exposure to PBS resulted in reduced surface free energy for both materials, with a significantly stronger effect observed for NiTi_Ta_2_O_5_, leading to a change from hydrophilic to hydrophobic surface characteristics.

**Figure 10:**
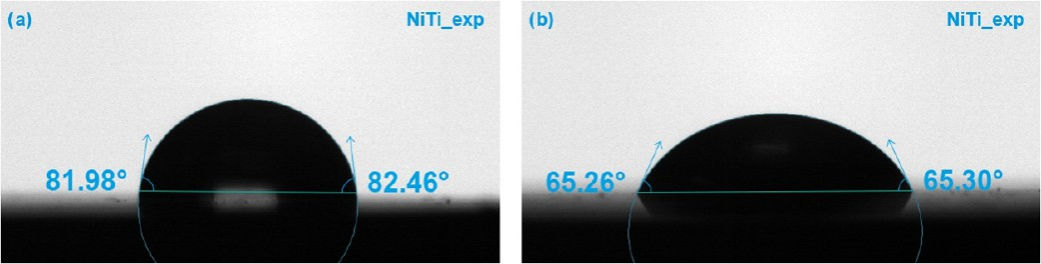
Comparative surface wettability analysis of NiTi after 60-day exposure to PBS: (a) water contact angle and (b) diiodomethane contact angle measurements.

**Figure 11:**
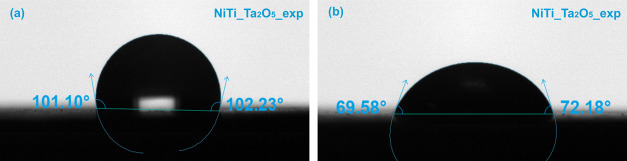
Comparative surface wettability analysis of NiTi-Ta_2_O_5_ after 60-day exposure to PBS: (a) water contact angle and (b) diiodomethane contact angle measurements.

**Table 2:** Results of Wettability and Surface Free Energy Measurements of the Substrate and Ta_2_O_5_ Coating in the Initial State and after Exposure

	Θ H_2_O [°]	Θ I_2_CH_2_ [°]	γ^d^ [mN/m]	γ^p^ [mN/m]	SFE [mN/m]
NiTi	54 ± 4	54 ± 1	32.08	19.37	51.45
NiTi_exp	83 ± 1	61 ± 3	27.78	4.92	32.69
NiTi_Ta_2_O_5_	69 ± 5	54 ± 2	32.13	10.04	42.17
NiTi_Ta_2_O_5__exp	104 ± 3	68 ± 3	23.74	0.42	24.16

### 3.8 Tribology

The highest average dry friction coefficient (TDF) value was observed for the NiTi_Ta_2_O_5_ sample. A decrease in the same value was observed for the NiTi_exp and NiTi_Ta_2_O_5__exp samples compared to the initial sample (NiTi) and NiTi_Ta_2_O_5_ before the exposition for solution. This means that the abrasion resistance of the samples after exposition has improved compared with the initial state. Tribological testing of the deposited Ta_2_O_5_ coating showed a relatively lower friction coefficient of *f* = 0.82, compared to the initial state, where the friction coefficient was *f* = 0.83 (Figure 12).

**Figure 12:**
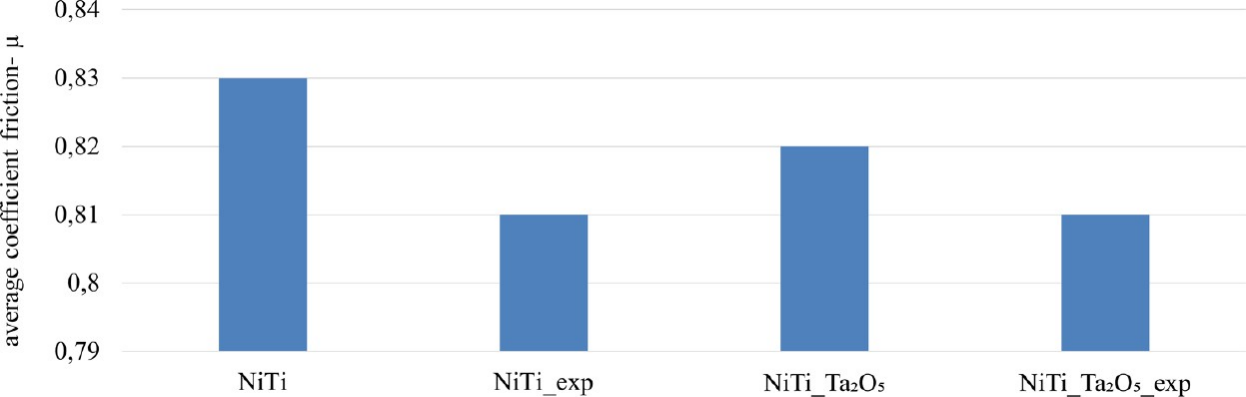
Average friction coefficient, technically dry friction (TDF).

In addition, mass loss measurements were conducted as part of the tribological tests. A summary of the results indicates differences in the mass loss among the tested samples. For the NiTi sample, the mass loss is 0.033, while for the NiTi_exp sample, it is lower at 0.023, indicating an improvement in the material’s resistance following soaking and exposure in the solution. For samples with a Ta_2_O_5_ coating, exposure to the solution did not affect the mass loss value. The results for the NiTi_Ta_2_O_5_ and NiTi_Ta_2_O_5__exp samples are comparable. In summary, the NiTi_exp sample exhibits the lowest mass loss and thus the best wear resistance despite exposure to the solution, while the highest mass loss is observed for NiTi_Ta_2_O_5_, indicating that coating does not improve the wear resistance in this case (Table 3).

**Table 3:** Mass Loss after the Wear Test

	before [g]	after [g]	mass loss [g]
NiTi	1.060	1.027	0.033
NiTi_exp	1.058	1.035	0.023
NiTi_Ta_2_O_5_	1.077	1.023	0.054
NiTi_Ta_2_O_5__exp	1.072	1.019	0.053

An analysis of the geometric structure of the surface was performed. The depth and friction surface determined based on the generated profile of the worn surface (the wear mark left after tribological testing) were taken as a measure of sample wear. There are obvious furrows and scratches, indicating abrasive wear and adhesive wear (Figure 13).

**Figure 13:**
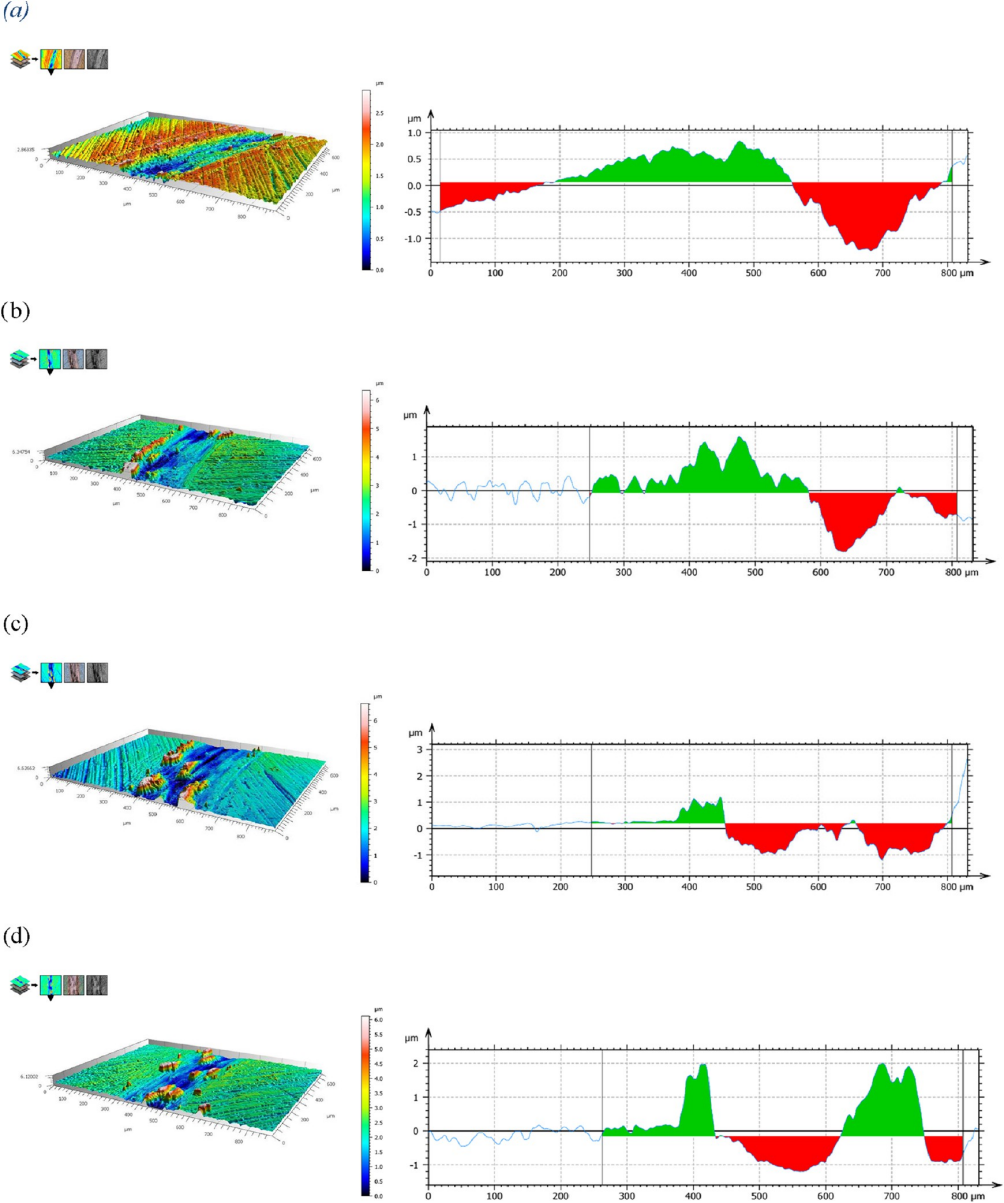
Isometric images of wear marks and wear profiles on a cross-section during dry friction TDF, (a) NiTi, (b) NiTi_exp, (c) NiTi_Ta_2_O_5_, and (d) NiTi_Ta_2_O_5__exp.

The coatings display a superslow scratch, which is confirmed by 3D (three-dimensional) morphology. The exposure of the samples caused a decrease in wear. The depth and width of the wear scar were diminished. The analysis of isometric images allowed the depth and friction area to be determined based on the generated worn surface profiles. The quantitative analyses of the wear volume (Figure 14). The parameter of wear depth was observed to be the same value for all of the samples, which confirms the appearance of the isometric image and the slight wear mark.

**Figure 14:**
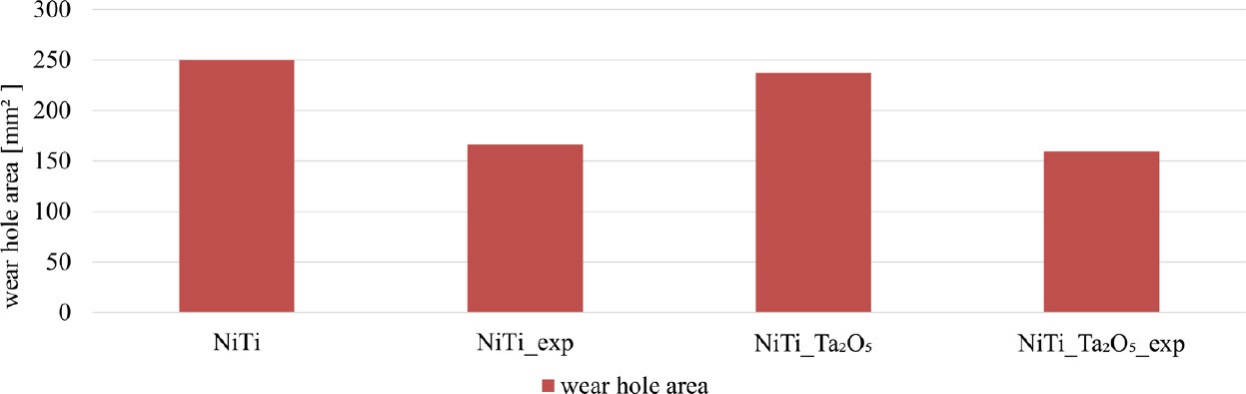
Average wear area on a cross-section.

In the case of the wear area assessment, the lowest wear area value was observed for the exposition samples, while the highest value during friction was achieved for the NiTi sample. This indicates that the nanocoating by Ta_2_O_5_ alleviates the wear behavior during the friction process compared to the NiTi sample. The exposition process enhances the wear resistance and load-bearing capacity during the friction test and indicates that they possess excellent antiwear properties after modification in comparison to shortage of surface modification. The expositions have the same influence for samples with and without modification (Figures 14,15).

**Figure 15:**
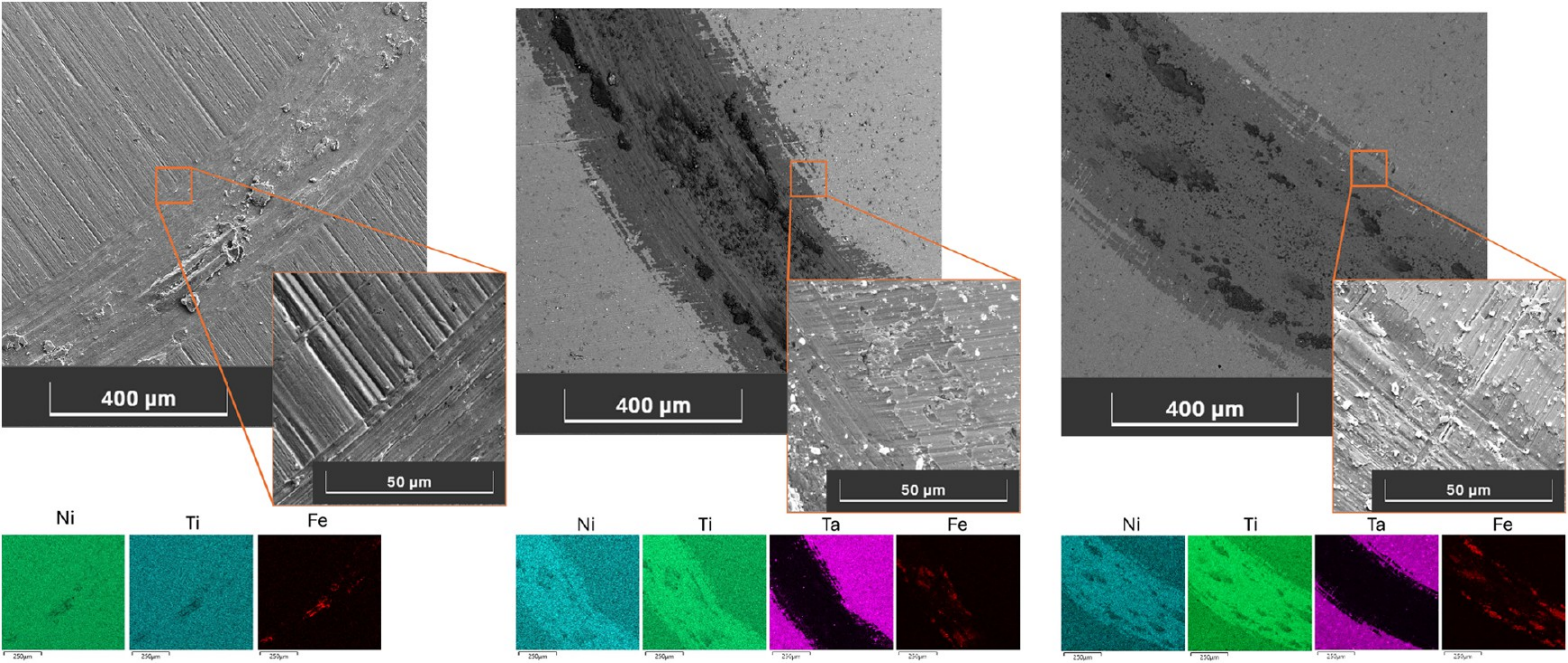
Example morphology of the abrasion surface resulting from tribological measurements with EDS analysis for (A) −NiTi_exp, (B) −NiTi_Ta_2_O_5_, and (C) −NiTi_Ta_2_O_5__exp.

Detailed SEM analysis of the wear marks revealed almost complete removal of the deposited Ta_2_O_5_ coating along the ball’s path (Figure 15). For uncoated samples, the friction edges were smooth and uniform, while the wear marks for coated samples exhibited a jagged, irregular structure, suggesting the brittle nature of the nanocoating. For each of the tested variants, the deposition of the ball material used during the measurements was demonstrated on the surface. The area of deposited iron for the coated material was significantly larger than for the uncoated material, which showed only a point-like presence of iron.

### 3.9 Corrosion Studies

The corrosion resistance of the NiTi alloy and surface-modified samples was assessed on the basis of electrochemical parameters: corrosion potential (*E*_corr_), corrosion current density (*i*_corr_), and polarization resistance (*R*_p_). The values are presented in Table 4 as averages with the standard deviation. Sample curves of the tested samples are shown in Figures 16 and 17.

**Figure 16:**
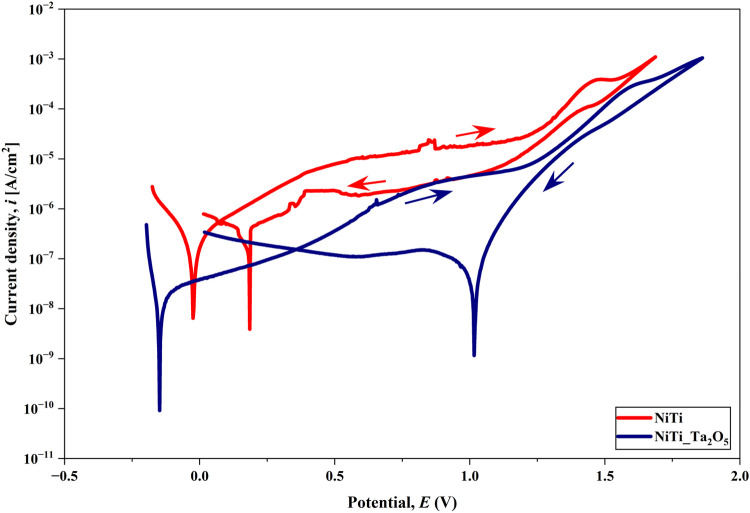
Example polarization curves for NiTi and NiTi with Ta_2_O_5_ coating samples.

**Figure 17:**
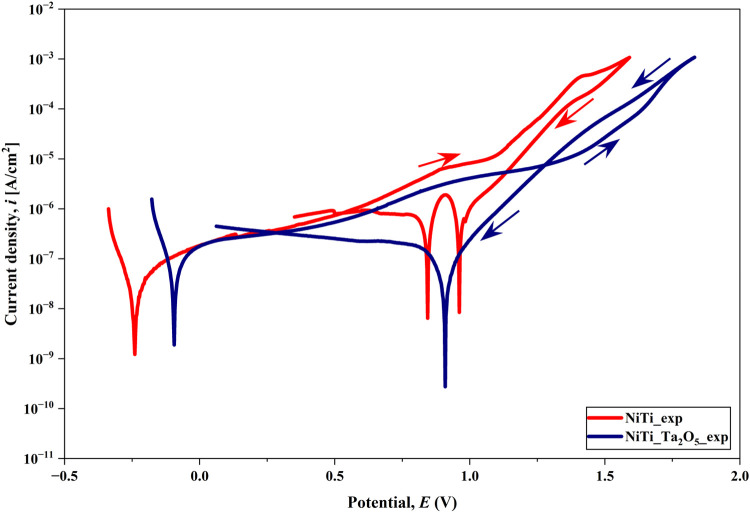
Example polarization curves for NiTi and NiTi with Ta_2_O_5_ coating samples after 60 days of incubation in PBS solution.

**Table 4:** Electrochemical Corrosion Parameters of Bare and Ta_2_O_5_-Coated NiTi Alloy before and after a 60-Day Exposure to PBS Solution

sample	*E*_corr_ [V]	*i*_corr_ [A/cm^2^]	*R*_*p*_ [Ω/cm^2^]
NiTi	–0.04 (±0.02)	2.40 × 10^–7^ (±5.56 × 10^–8^)	1.12 × 10^5^ (±2.46 × 10^4^)
NiTi_exp	–0.17 (±0.06)	1.15 × 10^–7^ (±8.30 × 10^–8^)	3.85 × 10^5^ (±3.57 × 10^5^)
NiTi_Ta_2_O_5_	–0.15 (±0.03)	7.63 × 10^–8^ (±4.62 × 10^–8^)	5.26 × 10^5^ (±4.65 × 10^5^)
NiTi_Ta_2_O_5__exp	–0.10 (±0.01)	7.60 × 10^–8^ (±1.10 × 10^–8^)	3.47 × 10^5^ (±4.87 × 10^4^)

The NiTi sample in its initial state was characterized by the highest corrosion current density (2.40 × 10^–7^ A/cm^2^) and the lowest polarization resistance (1.12 × 10^5^ Ω**/**cm^2^), which clearly indicates the highest rate of corrosion processes.

After 60 days of exposure to PBS solution, a nearly twofold decrease in *i*_corr_ (1.15 × 10^–7^ A/cm^2^) and a more than threefold increase in *R*_p_ (3.85 × 10^5^ Ω**/**cm^2^) were observed for the NiTi sample. The observed improvement in corrosion resistance after prolonged exposure in PBS solution, associated with natural passivation processes and stabilization of the oxide layer, may be further enhanced by phenomena occurring at the material–electrolyte interface. Observations of the sample surfaces after exposure showed the formation of calcium- and phosphorus-containing deposits, which aggregated over the entire surface in the form of a crystalline, locally cracked layer (Figure 16). Such precipitation products, originating from the PBS solution, can act as an additional physical diffusion barrier, limiting the access of the electrolyte to the substrate and slowing ion exchange. Although a significant portion of the sediment is removed during rinsing, the presence of a thin residual layer indicates permanent modification of the surface and may partially explain the reduction in corrosion current density.

The application of the Ta_2_O_5_ coating applied by the ALD method resulted in a further reduction of the corrosion current density to 7.63 × 10^–8^ A/cm^2^ and an increase in *R*_p_ to 5.26 × 10^5^ Ω**/**cm^2^. This means the lowest corrosion rate among all analyzed variants. The improvement in protective properties results from the presence of a Ta_2_O_5_ layer with high electrical resistivity, which limits both the transport of metal ions into the solution and the diffusion of oxygen and chloride ions into the substrate. After prolonged exposure to PBS, the electrochemical parameters changed only slightly. The *i*_corr_ value (7.60 × 10^–8^ A/cm^2^) remained virtually unchanged from before exposure, while *R*_p_ decreased to 3.47 × 10^5^ Ω/cm^2^. A slight decrease in polarization resistance may indicate partial hydration or local degradation of the coating, e.g., the formation of micropores or defects induced by prolonged exposure to the electrolyte. However, the lack of a significant increase in *i*_corr_ indicates that the continuity of the protective barrier has been largely maintained and the coating continues to effectively limit corrosion processes.

The cyclic potentiodynamic polarization curves obtained for uncoated NiTi and NiTi coated with Ta_2_O_5_ by the ALD method, both before and after 60 days of exposure to PBS solution, provide insights into the stability of passive films, their response to anodic polarization, and the ability of the systems to recover passivity.

In the as-deposited state, both materials exhibit a broad passive region characterized by relatively low current densities. For uncoated NiTi, this behavior is associated with the formation of a titanium oxide film, which provides inherent corrosion resistance. In the case of the Ta_2_O_5_-coated sample, the electrochemical response is additionally governed by the presence of the deposited tantalum oxide layer. This layer acts as a dielectric barrier and contributes not only to the limitation of ionic transport but also to capacitive current associated with charging and discharging processes at the oxide/electrolyte interface.^43^

The Ta_2_O_5_-coated sample generally exhibits current densities lower than those of bare NiTi, indicating improved corrosion resistance. This confirms the protective role of the coating, which effectively suppresses anodic dissolution. However, in both cases, the transition from passive behavior to higher current densities with increasing potential is gradual rather than abrupt. No clearly defined breakdown or pitting potential can be identified, and instead, the increase in current should be interpreted as progressive passive film destabilization occurring over a range of potentials around 1.2 V. This behavior suggests that the passive films do not undergo sudden catastrophic breakdown but rather experience distributed activation processes.

At higher anodic potentials, the increase in current density may also be partially associated with transpassive processes, including oxygen evolution resulting from water oxidation.^44^ Therefore, the observed current rise should not be attributed solely to localized corrosion processes but rather to a combination of passive film destabilization and electrochemical reactions occurring at elevated potentials.

A key feature of the as-deposited samples is the character of the reverse scan. In several regions, the current densities recorded during the reverse scan are lower than those observed during the forward scan at the same potentials, indicating the presence of negative hysteresis, Figure 16. This behavior suggests that the degree of surface passivation increases at more noble potentials, leading to enhanced stability of the passive film during the reverse scan. In other words, exposure to higher anodic potentials appears to promote the formation or restructuring of a more protective oxide layer. Such behavior is not characteristic of classical pitting corrosion but rather of systems exhibiting strong passivation and the ability to improve their protective properties under anodic polarization.^45^

After 60 days of exposure to PBS solution, both bare and Ta_2_O_5_-coated NiTi show modified electrochemical responses, indicating changes in the passive surface films due to a long-term interaction with the electrolyte. This can be attributed to the growth, thickening, and chemical stabilization of passive layers.^46^ Both variants maintain passive behavior after exposure, as evidenced by their relatively low current densities over a wide potential range. The general shape of the anodic branches remains similar to a gradual increase in current density at higher potentials and no clearly defined breakdown point. This again indicates progressive destabilization rather than abrupt passive film failure.

The reverse scans of the exposed samples confirm that both variants retain the ability to repassivate. A decrease in current density is observed after reversal of the scan direction, indicating recovery of the passive behavior. This demonstrates that even after prolonged exposure to PBS, the passive films are not irreversibly damaged and can be restored upon reduction of the potential. However, an important difference is observed for the Ta_2_O_5_-coated sample after exposure, where a small positive hysteresis loop appears in the high-potential region, Figure 17. This indicates that following passive film destabilization, the current density during the reverse scan remains temporarily higher than during the forward scan at the same potentials. Such behavior suggests the occurrence of localized activation processes and delayed repassivation. Nevertheless, the relatively small size of the hysteresis loop and the eventual decrease in the current density indicate that these processes are limited and reversible. This behavior is consistent with the formation of metastable localized sites, which do not develop into stable, propagating pits.^46^

Importantly, the Ta_2_O_5_-coated sample exhibits current densities lower than those of bare NiTi both before and after PBS exposure, confirming that the protective effect of the coating is maintained over time. Although the coated system may show earlier deviation from ideal passive behavior, this is likely related to the electrochemical characteristics of the oxide layer rather than to true deterioration of corrosion resistance.

It is also worth noting that despite the overall improvement in corrosion resistance after applying the Ta_2_O_5_ coating, the parameters obtained do not show as significant an improvement as previously reported for thinner layers obtained with fewer ALD cycles.^47,48^ In this work, the use of 800 deposition cycles, leading to an increase in coating thickness, did not translate into a proportional increase in barrier properties and in some cases resulted in their deterioration. This phenomenon can be explained by a change in the layer growth mechanism and the accumulation of internal stresses in thicker coatings. As the thickness increases, structural defects such as microcracks, porosity, discontinuities, or local delamination may appear, which facilitate electrolyte penetration and create preferential ion transport paths to the substrate.^49–51^ In addition, a higher number of process cycles increase the total exposure time of the sample to elevated temperatures and chemical reagents, which can lead to modification of the NiTi/Ta_2_O_5_ interfacial zone, deterioration of adhesion, or formation of a transition layer with reduced tightness. Consequently, despite the greater nominal thickness, the actual effectiveness of the anticorrosion barrier may be lower than in the case of thinner, more homogeneous coatings, where layers deposited with fewer cycles showed more favorable protective properties.

The results obtained indicate that the main mechanism for improving corrosion resistance is the barrier effect of the Ta_2_O_5_ coating, which limits the charge transport and ion diffusion between the substrate and the electrolyte. Long-term exposure to PBS does not cause a significant loss of its protective properties, confirming its potential for biomedical applications requiring long-term operation in a physiological environment.

## 4 Conclusions

In conclusion, the surface engineering of NiTi shape memory alloys via atomic layer deposition (ALD) of tantalum oxide (Ta_2_O_5_) represents a fundamental breakthrough in maintaining the interfacial integrity within aggressive biological environments. Comprehensive physicochemical, structural, and mechanical analyses conducted after a rigorous 60-day immersion in phosphate-buffered saline (PBS) under static “worst-case scenario” conditions demonstrate the unprecedented stability of the proposed architecture. Scanning electron microscopy confirmed that 800 ALD cycles yielded a highly conformal, defect-free dielectric barrier (∼65 nm-thick) that effectively homogenized the initial substrate topography by sealing processing-induced cracks and grooves. Crucially, X-ray diffraction established the exclusive formation of an amorphous Ta_2_O_5_ phase with no evidence of deleterious phase transformations or crystallographic damage to the NiTi substrate, which fully retained its B2 austenite structure. Despite localized microdelamination observed under extreme conditions, the coating maintained its global protective functionality. Prolonged physiological exposure triggered a unique, self-regulating thermodynamic response at the tissue–implant interface. The initially hydrophilic surface underwent a transition to a highly hydrophobic state, reaching a water contact angle of 104°. Fourier transform infrared spectroscopy and surface free energy analyses elucidated the governing mechanism: the high-energy oxide surface actively adsorbs adventitious hydrocarbons and stimulates the precipitation of mineralized calcium and phosphate deposits. This spontaneously formed adlayer effectively shields reactive polar sites, driving the surface energy toward a highly stable minimum. In parallel with these chemical modifications, the Ta_2_O_5_ layer radically enhanced the surface mechanics. Tribological assessments (ball-on-disc) demonstrated that the amorphous ALD barrier serves as an advanced load-bearing layer, significantly reducing the coefficient of friction compared to that of bare NiTi. Its superior abrasive wear resistance ensures structural integrity under mechanical loading, thereby minimizing the risk of releasing cytotoxic wear debris into the bloodstream. By achieving this stable, mineralized, and thermodynamically inert state, the Ta_2_O_5_ layer ultimately functions as a superior diffusion barrier. It fundamentally arrests the outward migration and localized leaching of nickel ions, ensuring absolute interfacial stability, even under stagnant physiological conditions. This ALD-driven strategy transcends traditional biomaterial protection paradigms. Through the synergy of morphological, chemical, crystallographic, and tribological properties, this approach represents a transformative step toward ensuring the long-term biocompatibility and stability of nitinol-based cardiovascular implants.

## Supplementary Material



## Data Availability

The datasets generated and analyzed during the current study are openly available in the Zenodo repository (10.5281/zenodo.18657006) and the RepOD repository (10.18150/QDPVM2)

## Notes

Detailed experimental procedures covering materials, reagents, and analytical instrumentation; scanning electron microscopy images of reference sample surface morphology; X-ray diffraction spectra characterizing the bare NiTi substrate; and supporting references (PDF)
