# Supplementary material for: Surface Engineering of NiTi via Ta2O5 ALD: Stability Assessment of Barrier Properties against Exposure to Simulated Physiological Solution
Source: ACS Appl Mater Interfaces. 2026 May 6;18(19):27264–80. doi: 10.1021/acsami.4c22646 (PMC13308887; doi:10.1021/acsami.4c22646)
Supplement: Supplementary file 1 [file am4c22646_si_001.pdf]

Supporting Information

Surface engineering of NiTi via Ta_2O_5 ALD: stability assessment of barrier properties against exposure to simulated physiological solution

Julia Kolasa [a,*], Anna Taratuta [a], Barbara Rynkus [a], Julia Lisoń-Kubica [a], Karolina Wilk [a], Ada Orłowska [a], Maciej Krzywiecki [b], Jerzy Bodzenta [b], Karolina Szawiraacz [c], Karla Čech Barabaszová [d], Sylva Holešová [d], Anna Ziębowicz [a], Roman Major [c], Przemysław Kurtyka [c], Marcin Staniek [e], Marcin Basiaga [a,*]

[a] Department of Biomaterials and Medical Device Engineering, Faculty of Biomedical Engineering, Silesian University of Technology, Roosevelta 40, 41-800 Zabrze, Poland

[b] Institute of Physics - Centre for Science and Education, Silesian University of Technology, Konarskiego 22B, 44-100 Gliwice, Poland

[c] Institute of Metallurgy and Materials Science, Polish Academy of Sciences, Reymonta 25, 30-059 Kraków, Poland

[d] Nanotechnology Centre, CEET, VŠB – Technical University of Ostrava, 17. listopadu 2172/15, 708 00 Ostrava, Czech Republic

[e] Department of Transport Systems, Traffic Engineering and Logistics, Faculty of Transport and Aviation Engineering, Silesian University of Technology, Krasińskiego 8, 40-019 Katowice, Poland

Corresponding Authors *E-mail addresses: julia.kolasa@polsl.pl, marcin.basiaga@polsl.pl

Table of Contents

Experiment Section **3**

Figure S1. Surface morphology of reference samples. A - NiTi, B – NiTi_exp. **4**

Figure S2. Characterization of NITi – XRD analyses: NiTi (bare material) showing slightly different composition, together with $NiTi_2$ and Ni_3Ti phases. **4**

References **5**

Experiment Section

Materials and reagents. NiTi sheets (Wolften Co. Wrocław, Poland), precursor ALD system - Tantalum(V) ethoxide (99.99%) PURATREM, 93-7303, LOT L02902011, Strem Chemicals. Nitrogen (N_2) gas of 5.0 purity, were supplied by SIAD (Poland), Isopropanol (2-propanol, analytical grade, C_3H_8O, M = 60.1 g/mol) used for ultrasonic cleaning. Diiodomethane (CH_2I_2, analytical grade, M = 267.84 g/mol, CAS: 75-11-6) and distilled water used for surface free energy (SFE) calculations were purchased from Shanghai Titan Technology Co., Ltd. (Shanghai, China). Steel balls (AISI 440-C, diameter 6 mm, $R_a \leq 0.05$ µm) used as counter-samples were provided by the tribometer manufacturer.

Instrumentation. Ultrasonic cleaning of the samples was performed using an ultrasonic device (Emmi-MF60, EMAG, Germany). Surface morphology and chemical composition analysis were obtained using a scanning electron microscope (SEM, TESCAN VEGA, Brno, Czech Republic) equipped with an Xplore energy dispersive spectroscopy (EDS) detector (Oxford Instruments, Oxford, UK). X-ray diffraction (XRD) tests were performed on a D8 Discover diffractometer (Bruker, USA) equipped with a LynxEye stripe detector. X-ray photoelectron spectroscopy (XPS) measurements were carried out using a multi-chamber experimental system (PREVAC, Poland) fitted with an EA15 hemispherical electron energy analyzer and a dual-anode XR-40B source. Fourier transform infrared (FT-IR) spectra were recorded by an FT-IR spectrometer (Nicolet iS50, Thermo Scientific, USA) using a Smart Orbit ATR accessory with a DTGS detector. Atomic force microscopy (AFM) analysis, including film thickness and stiffness measurements, was conducted using an XE-70 atomic force microscope (Park Systems, South Korea) with Tap300DLC and ContAl-G probes (Budget Sensors). Surface topography and microroughness parameters were measured by a Leica DCM 8 optical profilometer (Leica Microsystems, Germany) operating in confocal mode. Contact angle and surface free energy (SFE) were determined using an Attension Theta Flex optical tensiometer (Biolin Scientific, Sweden). Abrasive wear resistance was evaluated using an Anton Paar TRB tribometer (Anton Paar, Austria) in a ball-on-disc

configuration. Electrochemical corrosion studies were performed using an Autolab PGSTAT302N potentiostat (Metrohm, Netherlands) with Nova 2.1 software.

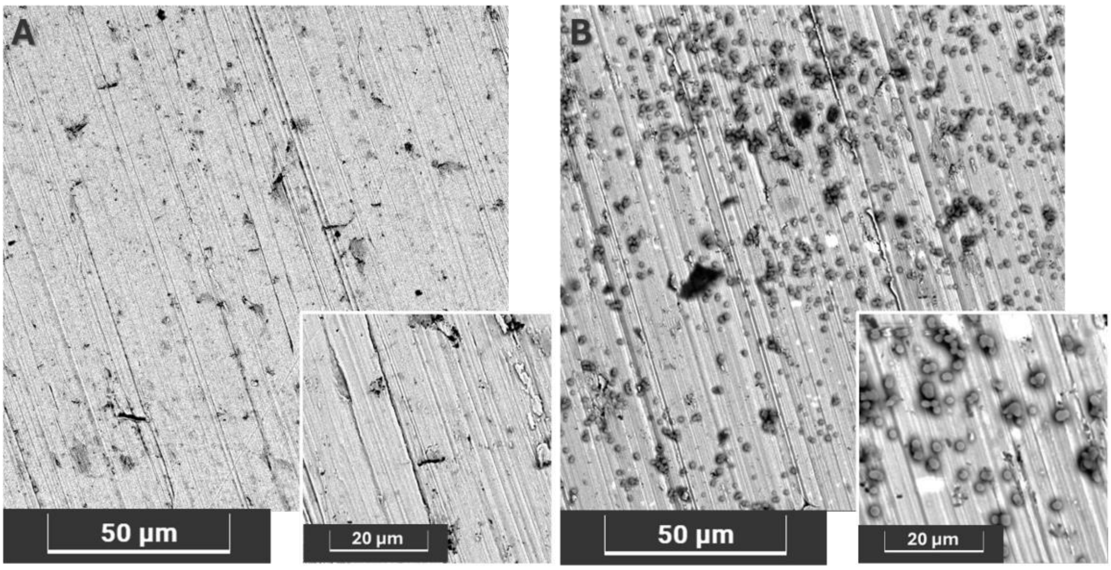

Figure S1. Surface morphology of reference samples. A - NiTi, B – NiTi_exp.

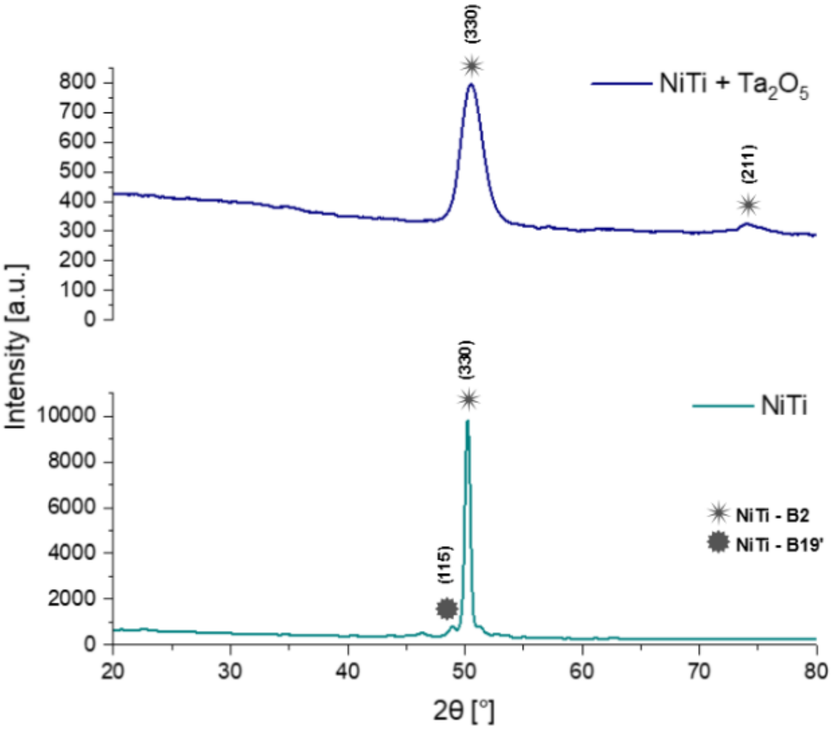

Figure S2. Characterization of NITi – XRD analyses: NiTi (bare material) showing slightly different composition, together with $NiTi_2$ and Ni_3Ti phases.

References

(1) Yang, H.; Yang, S.; Tang, M.; Li, B. The Electrosynthesis of Tantalum Ethoxide. *Electrochemistry* **2014**, *82* (9), 743–748. https://doi.org/10.5796/electrochemistry.82.743.

(2) Niskanen, A.; Kreissig, U.; Leskelä, M.; Ritala, M. Radical Enhanced Atomic Layer Deposition of Tantalum Oxide. *Chem. Mater.* **2007**, *19* (9), 2316–2320. https://doi.org/10.1021/cm0626482.

(3) Song, H.-J.; Koh, W.; Kang, S.-W. Atomic Layer Deposition of Ta2O5 Films Using Ta(OC2H5)5 and Nh3. *MRS Online Proceedings Library* **1999**, *567* (1), 469–471. https://doi.org/10.1557/PROC-567-469.

(4) Liang, X.; Lynn, A. D.; King, D. M.; Bryant, S. J.; Weimer, A. W. Biocompatible Interface Films Deposited within Porous Polymers by Atomic Layer Deposition (ALD). *ACS Appl. Mater. Interfaces* **2009**, *1* (9), 1988–1995. https://doi.org/10.1021/am9003667.

(5) Fabrication of Tantalum Oxide Layers onto Titanium Substrates for Improved Corrosion Resistance and Cytocompatibility. *Surface and Coatings Technology* **2015**, *272*, 58–65. https://doi.org/10.1016/j.surfcoat.2015.04.024.
